# Emergence of β-lactamase- and carbapenemase- producing *Enterobacteriaceae* at integrated fish farms

**DOI:** 10.1186/s13756-020-00736-3

**Published:** 2020-05-19

**Authors:** Dalia Hamza, Sohad Dorgham, Elshaimaa Ismael, Sherein Ismail Abd El-Moez, Mahmoud Elhariri, Rehab Elhelw, Eman Hamza

**Affiliations:** 1 Department of Zoonoses, Faculty of Veterinary Medicine, Cairo University, Giza square, PO Box 12211, Cairo, Egypt; 2 Department of Microbiology and Immunology, National Research Centre, Giza, Egypt; 3 Department of Veterinary Hygiene and Management, Faculty of Veterinary Medicine, Cairo University, Giza, Egypt; 4 Department of Microbiology, Faculty of Veterinary Medicine, Cairo University, Giza, Egypt

**Keywords:** Egypt, Aquaculture-agriculture-farms, *Enterobacteriaceae*, β lactam-resistance, Inc. plasmids, PBRT kits

## Abstract

**Background:**

Epidemiological studies suggested that determinants for antibiotic resistance have originated in aquaculture. Recently, the integrated agriculture-aquaculture system has been implemented, where fish are raised in ponds that receive agriculture drainage water. The present study aims to investigate the occurrence of β-lactamase and carbapenemase-producing *Enterobacteriaceae* in the integrated agriculture-aquaculture and the consequent public health implication.

**Methods:**

Samples were collected from fish, fishpond water inlets, tap water, outlet water, and workers at sites of integrated agriculture-aquacultures. Samples were also taken from inhabitants of the aquaculture surrounding areas. All samples were cultured on MacConkey agar, the *Enterobacteriaceae* isolates were tested for susceptibility to cephalosporins and carbapenems, and screened for *bla*_CTX-M-15_, *bla*_SHV_, *bla*_OXA-1_, *bla*_TEM_, *bla*_PER-1_, *bla*_KPC_, *bla*_OXA-48_, and *bla*_NDM_. Strains having similar resistance phenotype and genotype were examined for the presence of Incompatible (Inc) plasmids.

**Results:**

A major proportion of the *Enterobacteriaceae* isolates were resistant to cephalosporins and carbapenems. Among the 66 isolates from fish, 34 were resistant to both cephalosporin and carbapenem groups, 26 to carbapenems alone, and 4 to cephalosporins alone. Of the 15 isolates from fishpond water inlets, 8 showed resistance to both groups, 1 to carbapenems alone, and 5 to cephalosporins alone. Out of the 33 isolates from tap water, 17 were resistant to both groups, and 16 to cephalosporins alone. Similarly, of the 16 outlet water isolates, 10 were resistant to both groups, and 6 to cephalosporins alone. Furthermore, of the 30 examined workers, 15 carried *Enterobacteriaceae* resistant strains, 10 to both groups, and 5 to cephalosporins alone. Similar strains were isolated from the inhabitants of the aquaculture surrounding areas. Irrespective of source of samples, strains resistant to all examined antibiotics, carried predominantly the carbapenemase gene *bla*_KPC_ either alone or with the β-lactamase genes (*bla*_CTX-M-15_, *bla*_SHV_, *bla*_TEM_, and *bla*_PER-1_). The isolates from fish, water, and workers harboured a wide-range of multi-drug-resistance Inc. plasmids, which were similar among all isolates.

**Conclusion:**

The present findings suggest transmission of the resistance genes among *Enterobacteriaceae* strains from different sources. This reiterates the need for control strategies that focus on humans, animals, water, and sewage systems to solve the antibiotic resistance problem.

## Introduction

A link between aquaculture and the development of antibiotic resistance has been demonstrated by an increasing body of research [1, 2]. Worldwide, there is a massive increase in fish farming, which is associated with intensive use of antibiotics to combat bacterial infections [3]. Other potential explanations are contamination of aquaculture with antibiotic residues and antibiotic resistant bacteria coming from animals through using the so-called livestock integrated aquaculture system [4–6]. Recently, an integrated agriculture-aquaculture system has been implemented to save water resources [7]. In such system, fish are reared in ponds receiving water from crop farms through irrigation canals.

The major concern with the use of antibiotics in aquaculture is that fish do not effectively metabolize antibiotics and pass them largely unused in faeces [8]. This encourages the development of antibiotic resistance in bacteria present in fish and the surrounding environment [1, 2, 9]. Fishponds are not subject to frequent water exchange, resulting in accumulation of antibiotic-residues and -resistant bacteria, which are then disseminated at harvest time.

Fish bacteria particularly *Enterobacteriaceae* can exchange antibiotic resistance genes with human and animal bacteria [10, 11]. Among these, β-lactam resistant bacteria are of great concern, as they are becoming non-susceptible to nearly all available antibiotics [12, 13]. They include extended-spectrum β-lactam (ESBL) and carbapenem-resistant (CRE) *Enterobacteriaceae*. ESBL carry a broad spectrum of β*-*lactamase enzymes that hydrolyse a wide range of penicillin and cephalosporin antibiotics, but not carbapenems [14]. However, CRE carry carbapenemase enzymes that confer resistance to nearly all β-lactam antibiotics, including carbapenems [15]. The β*-*lactamase enzymes encompass members of the TEM and SHV families and other groups, such as CTX-M, OXA, and PER β-lactamases [16]. The carbapenemase enzymes are a diverse group of β*-*lactamases, the most remarkable of which are the big five enzymes KPC, NDM, OXA-48, IMP, and VIM [17]. There is strong evidence that the β*-*lactamase and carbapenemase encoding genes are found on plasmids that facilitate their transfer among bacteria of different genera and kingdoms [15, 16]. Plasmids are extra-chromosomal, self-replicating genetic elements, carried by the bacteria. Plasmids having the same origin of replication cannot stably co-reside within the same bacterial cells, are known as incompatible (Inc) [18]. The Inc. plasmids can positively select for the presence of resistance genes [19]. Identification of the Inc. plasmids in the bacterial isolates helps to trace the transfer of the resistance genes. Currently, a PCR method is accepted to classify the Inc. resistance plasmids based on their type of replicon [20]. Thirty replicons are now recognised, which represent the major replicase genes of the Inc. plasmids circulating among *Enterobacteriaceae*, including HI1, HI2, I1, I2, X1, X2, X3, X4, L, M, N, FIA, FIB, FIC, FII, FIIS, FIIK, FIB-KN, FIB-KQ, W, Y, P1, A/C, T, K, U, R, B/O, HIB-M, and FIB-M [21].

Recently in Egypt, small scale use of the integrated agriculture-aquaculture system began to be used. The aims of the present study are, therefore, to investigate the extent of antibiotic resistance in aquacultures integrated with agriculture in Egypt and its public health significance. Accordingly, this was performed through (i) assessing the prevalence of β-lactamase (βLPE) and carbapenemase-producing *Enterobacteriaceae* (CPE) in the water inlets of aquaculture originating from agriculture and supply fishponds, in fish, tap water, and the outlet water of the aquaculture; (ii) determination of the occurrence of βLPE and CPE in aquaculture workers and inhabitants of the aquaculture surrounding areas, those having a history of eating fish from these aquaculture and also use the same tap water source as the aquaculture (iii) identifying the presence and type of Inc. plasmids carried by the resistant strains isolated from different sources.

## Materials and methods

### Sample collection

**Fig. 1:**
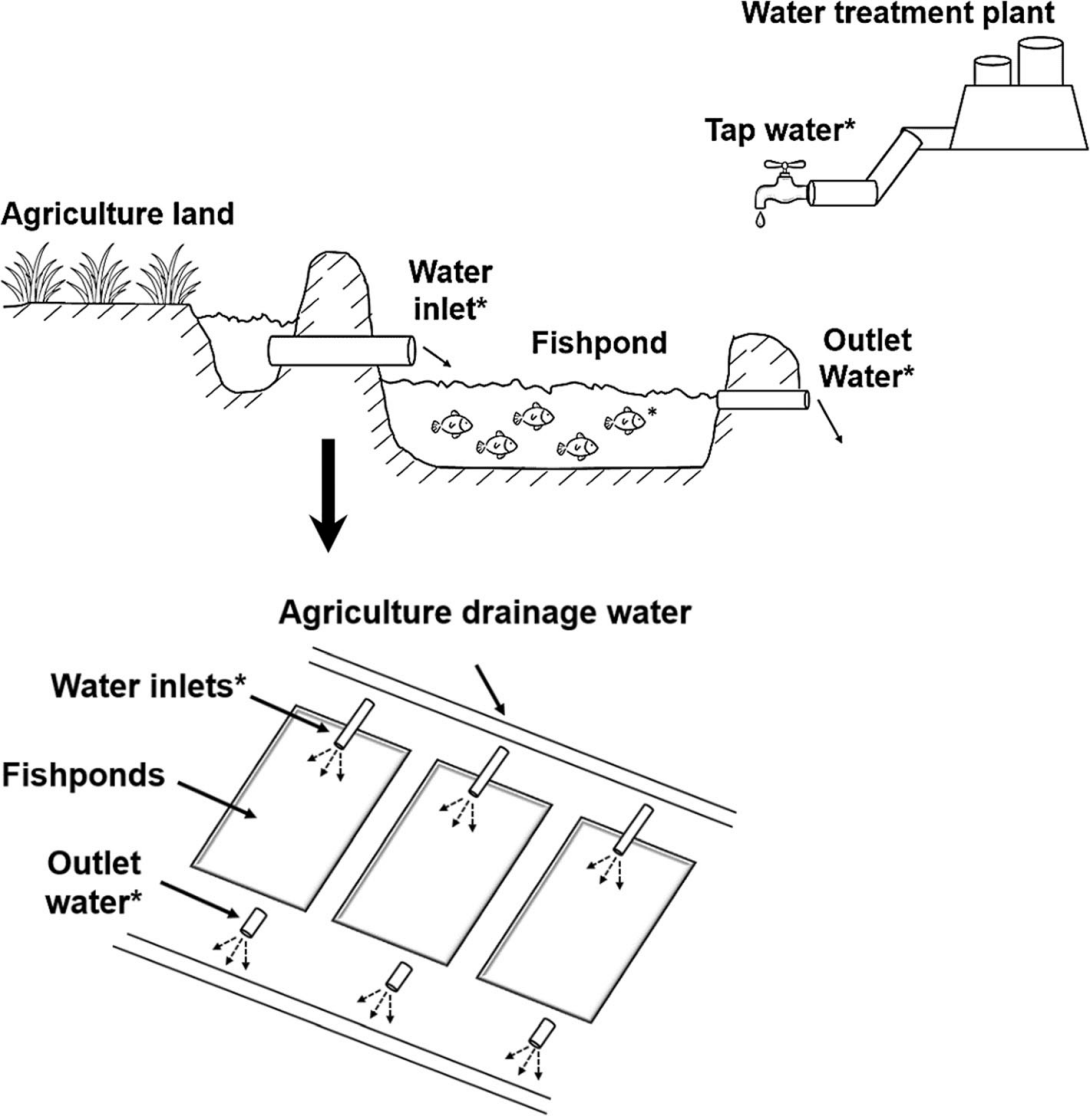
Illustration of the setup of the integrated agriculture-aquaculture farms included in the present study. Locations where samples were collected are indicated with an asterisk (*)

Four integrated agriculture-aquaculture farms were randomly selected from the Giza governorate, the third-largest city in Egypt. The selected farms are located near each other (approx. 5 km away from each other), they combine agriculture with fish farming As illustrated in Fig. 1, the fish are raised in ponds, each fishpond receives water from agriculture drainage water through a separate water pipe (Water inlet). Tap water is another source of water for the aquaculture, which is used to dissolve medicines given to the fish and sometimes as a supply for the fishponds in case of shortage of agriculture water. Tap water is also used by the workers for drinking and hand washing. Outlet water represents the waste drained from fishponds through pipes.

Samples were aseptically collected from internal organs (liver, spleen, and kidney) from 105 apparently healthy fresh Tilapia fish (*Oreochromis niloticus*). Additionally, samples were collected from fishpond water inlets (*n* = 30), tap water (*n* = 44), and outlet water (*n* = 26). The water samples were placed in sterile glass bottles containing sodium thiosulphate and were filtered through 0.4 μm pore size nitrocellulose filters (Sartorius, Aubagne, France) placed on tubes containing peptone broth [22].

Faecal and hand swab samples were collected from humans working in the aquaculture farms (*n* = 30), having contact with the fish, and who agreed to participate in the study.

Additionally, faecal samples were collected from inhabitants of the aquaculture surrounding areas (*n* = 45), who use the same source of tap water as the aquacultures and were admitted to the local medical centre with diarrhoea after eating fish from the tested aquacultures.

### Bacterial isolation and identification

All samples were inoculated into Trypticase soy broth tubes and were incubated at 37 °C for 24 h. A loop-ful from the previously incubated tubes were streaked onto MacConkey agar plates (Oxoid Ltd., Cairo, Egypt) and Eosin methylene blue agar (EMB) (Oxoid) and were incubated aerobically at 37 °C for 24 h. Suspected colonies were purified through subculture on MacConkey agar plates and were examined for morphology and phenotype traits according to Collee et al. [23].

### Biochemical identification

Pure colonies were subjected to API 20E kits (BioMerieux, Marcy-l’Étoile, France) according to the manufacturer’s instructions. The accuracy of API 20E results was 100% as determined by API web software (BioMérieux).

### Antibiotic susceptibility test

Confirmed strains of *Enterobacteriaceae* were examined for susceptibility to different antibiotics (Oxoid) using the disk diffusion test on Mueller-Hinton agar (Oxoid). The antibiotics included were cephalosporins [Cefoxitin (FOX, 30 μg), Ceftazidime (CAZ, 30 μg) Cefotaxime (CTX, 30 μg), Ceftriaxone (CRO, 30 μg)] and the carbapenems [Imipenem (IPM, 10 μg), Meropenem (MEM, 10 μg), Ertapenem (ETP, 10 μg)] groups. The results were interpreted according to the clinical breakpoints recommended by CLSI (Clinical laboratory Standard Institute) [24].

Based on CDC definitions [25], *Enterobacteriaceae* isolates resistant to at least one of the carbapenem drugs (Imipenem, Meropenem, Ertapenem, or Doripenem) or produce a carbapenemase gene are called carbapenem-resistant *Enterobacteriaceae* (CRE). Accordingly, in the present study, isolates resistant to at least one of the tested carbapenem drugs were considered CRE and were tested for the presence of carbapenemase genes.

### PCR for detection of β-lactamase- and carbapenemase- encoding genes

Genomic DNA from resistant isolates was extracted using a QIAamp® mini kit (Qiagen, Hombrechtikon, Switzerland). PCR was performed to identify β-lactamase-encoding genes (*bla*_CTX-M-15_, *bla*_SHV_, *bla*_TEM_, *bla*_PER-1_) using specific oligonucleotide primers as previously described [26]. The PCR for *bla*_OXA-1_ was done using specific oligonucleotide primers according to [27].

Multiplex PCR [28] was performed to identify carbapenemase-encoding genes (*bla*_KPC*,*_*bla*_OXA-48_, and *bla*_NDM_) using specific oligonucleotide primers [29]. The three genes were chosen to include one representative candidate from each of the three carbapenemase groups (Serine, OXA, and MLBs) and the selection was based on previous findings in Egypt [28]. The sequences of the primers used in the present study are shown in S1.

### Characterisation of Inc. plasmids present in the resistant isolates using PCR-based-replicon-typing

Genomic DNA from the isolates that were resistant to all tested antibiotics and showed similar resistance genotypes (*n* = 30) were subjected to plasmid typing using the PBRT 2.0 kit (PCR based replicon typing Ver.01/06/2017, Diatheva, Fano, Italy). The kit contains eight multiplex PCR, each of which amplifies three to four targets, allowing detection of a total of 30 replicons. It also includes positive control plasmids for the 8 multiplex PCR. The kit was used following the manufacturer’s instructions. Interpretation of the results was performed according to the manufacture instruction with IncF plasmids designated as multi-replicons when they showed a combination of FII replicons with FIA or FIB [30, 31].

### Statistical analysis

PASW Version 18 software (SPSS Inc., Chicago, IL, USA) was used for statistical analysis. Descriptive statistics were run and showed that the data are normally distributed. A Chi square (X^2^) test was used to determine whether there is a difference in the prevalence of the isolated *Enterobacteriaceae* strains and type of samples (fish, fishpond water inlets, tap water, and outlet water) (Table 1). Chi-square (X^2^) test was also performed to examine whether the prevalence of antibiotic resistance is influenced by the type of samples or species of bacteria, or both combined. On each occasion, the significance of differences was set at *p* value < 0.05.

**Table 1:** Prevalence of *Enterobacteriaceae* strains in Tilapia fish and water at four different aquacultures

Source (No. examined)	No. of ***Enterobacteriaceae***-positive samples			
	Total	***E. coli***	***ECC***	***K. pneumoniae***
**Fish (105)**	**66 (63%)**	45*	12	9
**Fishpond water inlets (30)**	**15 (50%)**	9*	4	2
**Tap water (44)**	**33 (75%)**	22*	6	5
**Outlet water (26)**	**16 (62%)**	12*	2	2

* indicates a significant difference in the prevalence of *E. coli* from that of *ECC* and *K. pneumoniae*

## Results

### Prevalence of *Enterobacteriaceae* in apparently healthy Nile tilapia fish and water from integrated agriculture-aquacultures

As demonstrated in Table 1, of the 105 tested apparently healthy Nile tilapia fish, 66 (63%) showed the presence of *Enterobacteriaceae*. Additionally, the occurrence of *Enterobacteriaceae* was also high among the collected samples from fishpond water inlets (15/30; 50%), tap water (33/44; 75%), and outlet water (16/26; 62%) from the aquacultures. *E. coli* represented the major species of *Enterobacteriaceae* isolated from fish and water. *Enterobacter cloacae complex (ECC)* and *Klebsiella (K.) pneumoniae* were also detected, but with a significantly lower prevalence than *E. coli*.

### Occurrence of cephalosporin- and carbapenem-resistant *Enterobacteriaceae* in the integrated agriculture-aquacultures

**Fig. 2:**
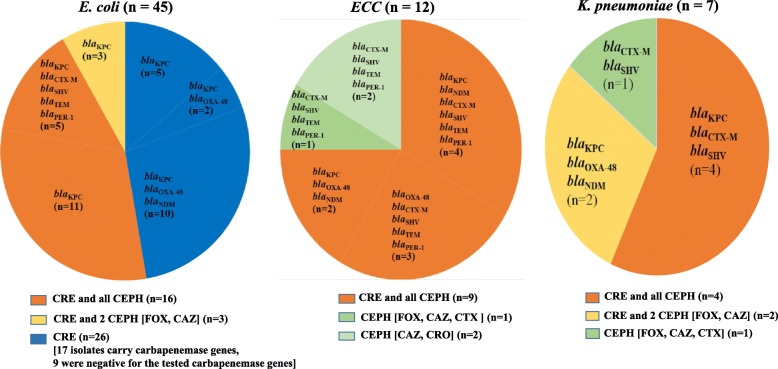
Number of carbapenem- and cephalosporin*-* resistant *Enterobacteriaceae* isolates among fish. The *Enterobacteriaceae* isolates from fish (*n* = 66), including *E. coli* (*n* = 45), *ECC* (*n* = 12), and *K. pneumoniae* (*n* = 9) were screened for resistance against carbapenems (CRE) and cephalosporins (CEPH: FOX, CAZ, CTX, and CRO). The strains are grouped according to their resistance phenotypes as follow: CRE and all CEPH (isolates resistant to at least one of the tested carbapenems and all tested cephalosporins); CRE and 2 CEPH (isolates resistant to at least one of the tested carbapenems and two of the tested cephalosporins); CEPH (isolates resistant to 2 or more of the tested cephalosporins but not to carbapenems); CRE (isolates resistant to at least one of the tested carbapenems but not to any of the tested cephalosporins). Each phenotype is marked with colour and its resistance genotypes are also provided; carbapenemase (*bla*_KPC_, *bla*_OXA-48_, and *bla*_NDM_) and β-lactamase (*bla*_CTX-M-15_, *bla*_SHV_, *bla*_OXA-1_, *bla*_TEM_, and *bla*_PER-1_) genes. (n) represents numbers of resistant strains

**Fig. 3:**
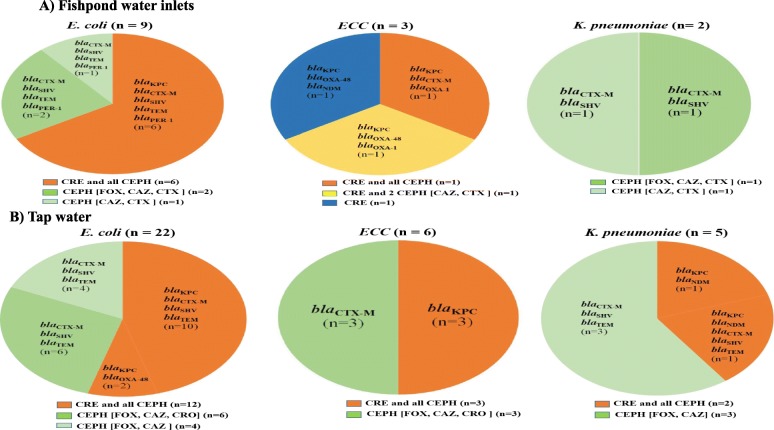
Number of carbapenem- and cephalosporin*-* resistant *Enterobacteriaceae* isolates among fishpond water inlets and tap water. The *Enterobacteriaceae* isolates from **a** fishpond water inlets (*n* = 14), including *E. coli* (*n* = 9), *ECC* (*n* = 3), and *K. pneumoniae* (*n* = 2), as well as from **b** tap water (*n* = 33), including *E. coli* (*n* = 22), *ECC* (*n* = 6), and *K. pneumoniae* (*n* = 5) were screened for resistance against carbapenems (CRE) and cephalosporins (CEPH: FOX, CAZ, CTX, and CRO). The strains are grouped according to their resistance phenotypes as follow: CRE and all CEPH (isolates resistant to at least one of the tested carbapenems and all tested cephalosporins); CRE and 2 CEPH (isolates resistant to at least one of the tested carbapenems and two of the tested cephalosporins); CEPH (isolates resistant to 2 or more of the tested cephalosporins but not to carbapenems); CRE (isolates resistant to at least one of the tested carbapenems but not to any of the tested cephalosporins). Each phenotype is marked with colour and its resistance genotypes are also provided; carbapenemase (*bla*_KPC_, *bla*_OXA-48_, and *bla*_NDM_) and β-lactamase (*bla*_CTX-M-15_, *bla*_SHV_, *bla*_OXA-1_, *bla*_TEM_, and *bla*_PER-1_) genes. (n) represents numbers of resistant strains

**Fig. 4:**
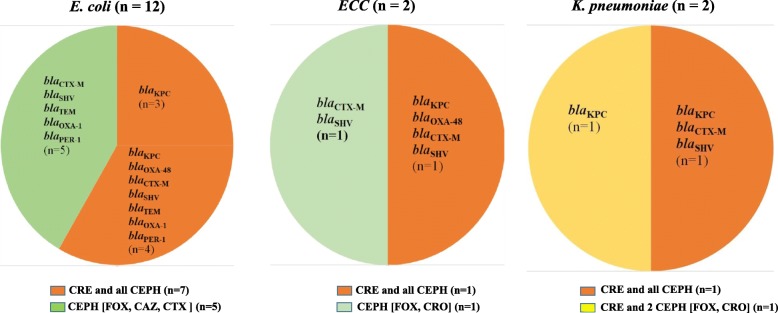
Number of carbapenem- and cephalosporin*-* resistant *Enterobacteriaceae* isolates among outlet water. The *Enterobacteriaceae* isolates from outlet water (*n* = 16), including *E. coli* (*n* = 12), *ECC* (*n* = 2), and *K. pneumoniae* (*n* = 2) were screened for resistance against carbapenems (CRE) and cephalosporins (CEPH: FOX, CAZ, CTX, and CRO). The strains are grouped according to their resistance phenotypes as follow: CRE and all CEPH (isolates resistant to at least one of the tested carbapenems and all tested cephalosporins); CRE and 2 CEPH (isolates resistant to at least one of the tested carbapenems and two of the tested cephalosporins); CEPH (isolates resistant to 2 or more of the tested cephalosporins but not to carbapenems). Each phenotype is marked with colour and its resistance genotypes are also provided; carbapenemase (*bla*_KPC_, *bla*_OXA-48_, and *bla*_NDM_) and β-lactamase (*bla*_CTX-M-15_, *bla*_SHV_, *bla*_OXA-1_, *bla*_TEM_, and *bla*_PER-1_) genes. (n) represents numbers of resistant strains

The *Enterobacteriaceae* isolates from fish and water samples of the aquacultures were screened for resistance against carbapenems (CRE) and cephalosporins (CEPH). The resistant strains were examined for carbapenemase and β-lactamase genes (Figs. 2*,*3, 4).

The Chi-square test showed that resistance to carbapenems and CEPH was significantly influenced by the source of samples (fish or water), but not by species of bacteria (*E. coli*, *ECC*, and *K. pneumoniae*). Indeed, Figs. 2, 3, 4 demonstrated that the resistance phenotype was similar between the three species of bacteria.

As displayed in Figs. 2, 3, 4, the isolates were grouped according to the resistance phenotype into; CRE and all CEPH (isolates resistant to at least one of the tested carbapenems and all tested cephalosporins); CRE and 2 CEPH (isolates resistant to at least one of the tested carbapenems and two of the tested cephalosporins); CRE (isolates resistant to at least one of the tested carbapenems, but not to any of the tested cephalosporins); CEPH (isolates resistant to 2 or more of the tested cephalosporins, but not to carbapenems).

Of the 66 fish isolates, 64 (45 *E. coli*, 12 *ECC*, 7 *K. pneumoniae*) showed resistance as follow; 29 to carbapenems and all tested cephalosporins, 5 to carbapenems and two of the tested cephalosporins, 26 to carbapenems alone, and 4 to cephalosporins alone (Fig. 2). Additionally, of the 15 isolates from fishpond water inlets, 7 were resistant to carbapenems and all cephalosporins, 1 to carbapenems and two cephalosporins, 1 to carbapenems alone, and 5 to cephalosporins alone (Fig. 3a). All the 33 isolates from tap water were resistant. Among them, 17 were resistant to carbapenems and all cephalosporins, and 16 were resistant to cephalosporins alone (Fig. 3b). Similarly, the 16 outlet water isolates were resistant, 9 to carbapenems and all cephalosporins, 1 to carbapenems and two cephalosporins drugs, and 6 to cephalosporins alone (Fig. 4).

Irrespective of the source of samples or species of bacteria, we found that strains resistant to all examined antibiotics, carry predominantly *bla*_KPC_ either alone or with the β-lactamase genes (*bla*_CTX-M-15_, *bla*_SHV_, *bla*_TEM_, *bla*_OXA-1_, and *bla*_PER-1_). However, strains resistant to carbapenems and two cephalosporins harbour only carbapenemase genes (*bla*_KPC_, *bla*_OXA-48_, *bla*_NDM_). Of the 26 fish isolates resistant only to carbapenems, 17 were carbapenemase producing, while 9 were negative to the three screened carbapenemase genes (Figs. 2, 3, 4).

### High numbers of aquaculture workers carried cephalosporin- and carbapenem-resistant *Enterobacteriaceae*

**Fig. 5:**
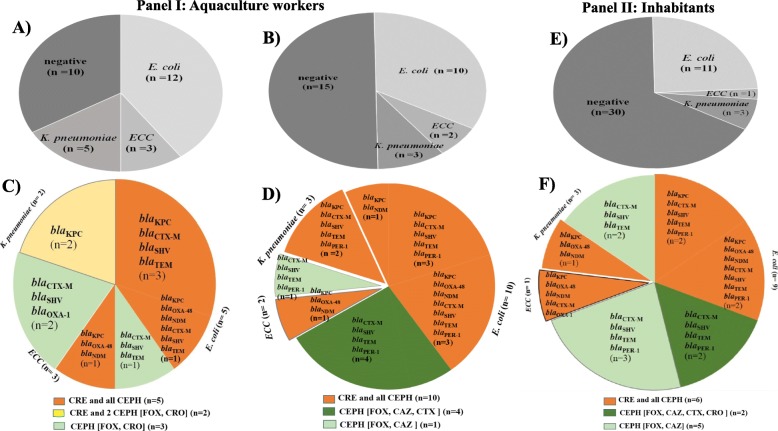
Number of cephalosporins- and carbapenems-resistant *Enterobacteriaceae* isolates from aquaculture workers and inhabitants of aquaculture surrounding areas. Panel I: Hand swab and faecal samples from aquaculture workers (*n* = 30) were examined. **a** and **b** illustrate the number (n) and species of *Enterobacteriaceae* isolated from hand swabs and faecal samples, respectively. **c** and **d** illustrate the number (n) of resistant *Enterobacteriaceae* isolates from hand swabs and faecal samples, respectively. Panel II: Faecal samples from inhabitants (*n* = 45) were examined. **e** illustrates the number (n) and species of *Enterobacteriaceae* isolated. **f** shows the number (n) of resistant *Enterobacteriaceae*. The strains are grouped based on resistance against carbapenems (CRE) and cephalosporins (CEPH: FOX, CAZ, CTX, and CRO) as follow: CRE and all CEPH (isolates resistant to at least one of the tested carbapenems and all tested cephalosporins); CRE and 2 CEPH (isolates resistant to at least one of the tested carbapenems and two of the tested cephalosporins); CEPH (isolates resistant to 2 or more of the tested cephalosporins but not to carbapenems). Each phenotype is marked with colour and its resistance genotypes are also provided; carbapenemase (*bla*_KPC_, *bla*_OXA-48_, and *bla*_NDM_) and β-lactamase (*bla*_CTX-M-15_, *bla*_SHV_, *bla*_OXA-1_, *bla*_TEM_, and *bla*_PER-1_) genes. (n) represents numbers of resistant strains

Hand swab and faecal samples were collected from humans working in the integrated agriculture-aquacultures. A relatively high proportion of the workers showed the presence of *Enterobacteriaceae* in hand swabs (20 / 30) and faecal samples (15 / 30) (Fig. 5, Panel I). Like in fish and water isolates, *E. coli* was the predominant species isolated from the human samples (Panel I A and B). Among the 20 isolates from hand swabs, 5 were resistant to carbapenems and all cephalosporins, 2 to carbapenems and two of the tested cephalosporins, and 3 to cephalosporins alone (Panel I C). Of the 15 faecal isolates, 10 were resistant to carbapenems and all cephalosporins, and 5 to cephalosporins alone (Panel I D). Additionally, the resistance phenotype and genotype were similar among *E. coli*, *ECC*, and *K. pneumoniae.* Strains resistant to all examined antibiotics harboured predominantly *bla*_KPC_ either with the other carbapenemase genes (*bla*_NDM_ and *bla*_OXA-48_) or with the β-lactamase genes (*bla*_CTX-M-15_, *bla*_SHV_, *bla*_TEM_, and *bla*_PER-1_) (Panel I C and D).

Faecal samples were also collected from inhabitants of the aquaculture surrounding areas. *Enterobacteriaceae* was isolated from 15 of the examined 45 samples with a predominance of *E. coli* (Fig. 5*,* Panel II A). Of the 15 isolates, 6 were resistant to carbapenems and all cephalosporins, and 7 were resistant to cephalosporins alone (Fig. 5, Panel II B). Consistent with the results of the aquaculture workers, the strains from the inhabitants were resistant to carbapenems and all cephalosporins carried predominantly *bla*_KPC_ with other carbapenemase genes (*bla*_NDM,_*bla*_OXA-48_) or with the β-lactamase genes (*bla*_CTX-M-15_, *bla*_SHV_, *bla*_TEM_, and *bla*_PER-1_) (Fig. 5, Panel II B).

### Similar types of incompatible (Inc) plasmids were carried by the *Enterobacteriaceae* resistant strains isolated from different sources as determined by the PBRT-kits

**Fig. 6:**
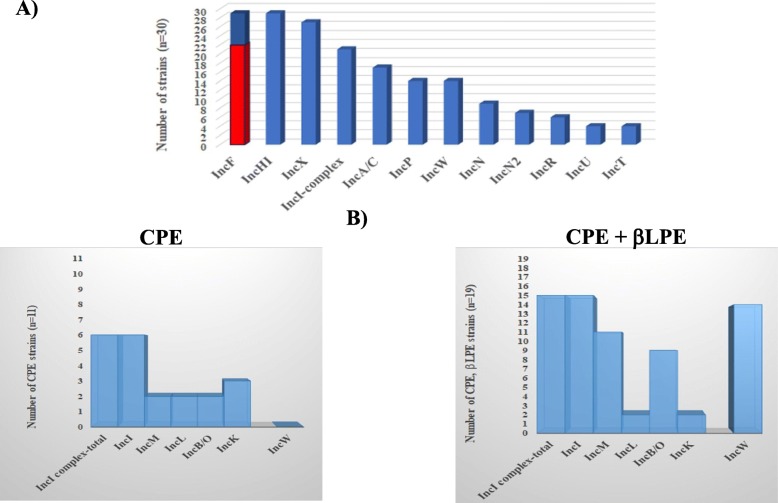
Types of Incompatible (Inc) plasmids carried by the *Enterobacteriaceae*-resistant isolates. **a** illustrates the types of Inc. plasmids found in the examined CRE isolates (*n* = 30) that were resistant to all tested CEPH and showed similar genotypes. The red colour in the histogram of the IncF plasmid indicates number of isolates that harbour multi-replicon. **b** shows the differences between the CRE isolates producing-carbapenemase genes alone (CPE, *n* = 11) and those producing both carbapenamese and β-lactamase (CPE + βLPE, *n* = 19) genes, in the proportion and types of Inc. plasmids

CRE isolates that were resistant to all tested antibiotics and showed similar resistance genotypes were examined for the presence and type of Inc. plasmids using PCR-Based-Replicon-Typing (S2, Fig. 6).

There were no differences in the type of Inc. plasmids, based on the source of the isolates (fish, fishpond water inlets, tap water, outlet water, and workers) (S2). As demonstrated in Fig. 6a, of the 30 examined resistant strains, 29 showed the presence of the IncF and the IncHI groups of plasmids, 28 carried the IncX, 21 harboured the IncI complex group, 17 had the epidemic plasmid IncA/C, and 14 carried the wide-host range IncP and the IncW groups. Other Inc. plasmids found in minority of the isolates were as follows; IncN (*n* = 9), IncN2 (*n* = 7), IncR (*n* = 5), IncU (*n* = 4), and IncT (*n* = 4).

It is important to note that, the majority of the IncF plasmids present (22 / 29) were multi-replicon (Fig. 6a), carrying the FII with FIA and or FIB replicons (S2) [30, 31]. A divergence in FIB (FIB-KN and FIB-KQ) and FII (FIIS and FIIK) replicons was observed. The FIB-KN was mainly found in the *K. pneumoniae* isolates, while the FIB-KQ was present in *K. pneumoniae* and *E. coli* isolates. Moreover, the FIIS and FIIK divergences were found in *E. coli*, *K. pneumoniae*, and *ECC* isolates (S2).

Some differences were noticed in the type of Inc. plasmids between strains producing carbapenemase genes alone (CPE) and those producing both carbapenemase and β-lactamase genes (CPE + βLPE) (Fig. 6b). Among them, the IncI complex plasmid was found in the higher proportion of the CPE + βLPE strains (15/19) than the CPE strains (6/11). The IncI complex contains five groups IncI, IncL, IncM, IncK, and IncB/O combined, or at least two of them. Another difference was the presence of the IncW plasmid only in CPE + βLPE strains (14 /19).

## Discussion

For the past 60 years, β-lactam antibiotics have been amongst the most successful drugs used for the treatment of bacterial infections in humans, animals, and fish [32–34]. In the current study, we investigated the occurrence of β-lactamase (βLPE)- and carbapenemase (CPE)- producing *Enterobacteriaceae* in fresh fish, fishpond water inlets, tap water, outlet water, and workers at four integrated agriculture-aquacultures. Additionally, faecal samples were collected from inhabitants of the aquaculture surrounding areas. The prevalence of *Enterobacteriaceae* isolated from apparently healthy fresh Nile Tilapia fish was 63%, which is higher than that reported by previous studies performed on fish raised in earthen pond in Egypt (44–53%) [35–37]. Like in fish, the aquaculture water showed a high prevalence of *Enterobacteriaceae*, particularly the tap water (75%) as compared to fishpond water inlets (50%) and outlet water (62%). Furthermore, of the 30 examined workers, 15 showed the presence of *Enterobacteriaceae* in the hand and faecal samples, whereas, 5 had *Enterobacteriaceae* only in the hand swabs. The species of *Enterobacteriaceae* isolated from fish, water samples and the workers included *E. coli*, *ECC*, and *K. pneumoniae*, with *E. coli* being the predominant species. Although the species of *Enterobacteriaceae* found could be fish pathogens [35–40], it is not the most common found bacteria in fish [2]. This together with the high prevalence of *Enterobacteriaceae* found in water, suggests faecal contamination in the aquaculture environment.

A high proportion of the *Enterobacteriaceae* isolates from the aquaculture showed resistance to the tested antibiotics, as found in fish (64 / 66), water inlets (14 / 15), tap water (33 / 33), outlet water (16 /16), and the workers (15 / 20). Regardless of the source of samples, a major number of these isolates were CRE, which encompass predominantly strains resistant to carbapenems and all tested cephalosporins, followed by strains resistant to carbapenems and some cephalosporins, while a few strains were resistant to carbapenems alone. The three phenotypes of CRE isolates had different resistance genotypes. However, CRE isolates resistant to all cephalosporins carried predominantly both carbapenemase and β-lactamase genes, CRE isolates resistant to some cephalosporins or to carbapenems alone harboured only carbapenemase genes. According to CLSI 2020, CRE isolates that produce one or more carbapenemase genes usually test resistant to one or more of cephalosporins, however, some CPE isolates can still test susceptible to cephalosporins [41]. This can be a possible explanation for our finding of CRE isolates which carry one or more of the carbapenemase genes (*bla*_KPC_, *bla*_OXA-48_, *bla*_NDM_), but susceptible to the tested cephalosporins (Figs. 2*,*3). In this regard, a study from South Africa also found some CPE-CRE isolates which were susceptible to cephalosporins [42].

It is important to note that among the 26 CRE fish isolates that showed resistance only to carbapenems, 9 isolates did not carry any of the tested carbapenemase genes, suggesting that they might harbour other carbapenemases which were not screened in the present study.

*Enterobacteriaceae* isolates resistant to cephalosporins alone were also found but in a lower proportion than the CRE. Slight differences were noticed between the four examined aquacultures, in the numbers of CRE and cephalosporins-resistant *Enterobacteriaceae*, which were not statistically different. Cephalosporins-resistant *Enterobacteriaceae* isolates have previously been found in fish and aquatic environments from different countries, including Egypt [40, 43–48]. However, a few recent studies showed the presence of CRE strains among fish [49, 50], indicating an increase in the prevalence of CRE in aquaculture. Irrespective of the species of bacteria or source of samples, the carbapenemase-producing isolates showed a predominance of *bla*_KPC_ over the other two genes *bla*_OXA-48_ and *bla*_NDM_, which agrees with other study that detected the presence of *bla*_KPC_ in seafood isolates [51]. Furthermore, all the isolates that were positive for β-lactamase genes harboured *bla*_CTX-M-15_ in combination with one or more of the other β-lactamase genes (*bla*_OXA-1_, *bla*_SHV_, *bla*_TEM,_ and *bla*_PER-1_). This is consistent with findings that showed a predominance of *bla*_CTX-M-15_ among β-lactam-resistant *Enterobacteriaceae* isolated from wild fish in Algeria [44], from frozen Mackerel in Saudi Arabia [45], and water collected from fish farms in Egypt [46]. Moreover, the *bla*_CTX-M_ was shown to be commonly associated with *bla*_TEM_ on the same plasmid [52], and possibly with *bla*_SHV_ and the broad spectrum *bla*_OXA-1_ [15, 51]. Our findings then raised the question of how such CRE strains have reached the aquacultures. The most likely explanation is a co-resistance to carbapenems by selective pressure from overuse of other antibiotics, for which resistance determinants are co-localised with carbapenemase on the same plasmid [53]. Another possible explanation might be unrestricted use of carbapenems to treat fish, which is consistent with our results that strains resistant only to carbapenems were found mainly among fish isolates. Unfortunately, this cannot be clarified, as no data is available on quantities and types of antibiotics used in aquaculture in Egypt. Furthermore, the resistant strains might access the aquaculture from agricultural sources, where animal manure is known to be used as fertilizers [6, 54, 55]. CRE has been isolated from dairy farms in Egypt [56]. Another possible source of the resistant strains could be tap water, which showed the presence of high amounts of CRE and cephalosporins-resistant *Enterobacteriaceae*, indicating faecal contamination. The main source of tap water in Egypt [57] is from surface water like rivers, lakes or canals. In many Egyptian governorates, sewage is usually discharged untreated into the surface water [58, 59]. Since sewage act as a pool of antibiotic residues as well as resistant bacteria from different sources [58, 59], this can result in contamination of tap water. This is supported by our results that showed the occurrence of *Enterobacteriaceae* in faecal samples from inhabitants of the aquaculture surrounding areas, which have similar resistance phenotype and genotype as the isolates from the aquaculture workers with a predominance of *bla*_KPC_ and *bla*_CTX-M-15_. In this regard, previous studies reported preponderance of *Enterobacteriaceae* carrying *bla*_KPC_ and *bla*_CTX-M-15_ among humans in Middle East [60] and North Africa [61, 62].

Characterisation of the Inc. plasmid types harboured by the CRE isolates that were resistant to all tested cephalosporins, revealed a similar profile among the isolates from fish, water, and workers. The major plasmid groups found were as follow; IncF, IncHI, IncX, IncI-complex, IncA/C, IncP, and IncW, which agrees with findings that IncF, IncHI, IncX, IncI-complex, and IncA/C are commonly associated with CRE isolated from humans and animals [19–21]. Crucially, the IncA/C is considered as an epidemic plasmid, as it has been detected in different countries and has a wide-host range of different sources [63]. IncP and IncW have been categorised among rarely reported plasmids [21, 64], however, we found them in a relatively high number of the isolates from different sources. Since these two plasmids have a broad-host range and can carry multiple resistance genes [64, 65], their presence in our isolates indicates a role in the spread of resistance genes among bacteria of different genera and sources. Other plasmids like IncN, IncN2, IncL, IncU, IncT, and IncR were also present, but in minority of the current isolates. This is consistent with [19], who stated that these plasmids are not often reported.

One of the interesting results is a predominance of the multi-replicon IncF among the tested CRE isolates, with divergence in FII (FIIS, FIIK) and FIB (FIB-KQ, FIB-KN), but not in FA (S2), suggesting that such plasmids are rapidly emerging with the subsequent increase in dissemination of resistance genes. IncF plasmid is unique, as it can carry many replicons, one replicon is strongly conserved for plasmid replication, while the others are free to diverge as a mechanism of evolution [19, 30]. Additionally, the IncF plasmid can co-integrate with other plasmids found in the current study, like IncI1, IncA/C, and IncN forming a mega-plasmid with multi-resistance regions [19, 31, 66, 67]. Taken together, the current CRE isolates carry a wide range of Inc. plasmids which are known to be associated with multi-drug resistance determinants. The profile of these plasmids was similar between isolates from fish, water, and humans working in contact with fish, suggesting a possible transfer among bacteria from different sources. To our knowledge, no data is available regarding the prevalent type of Inc. plasmids in Egypt.

## Conclusion

The present study reveals the occurrence of CRE which are resistant to all tested cephalosporins among fish, water, and workers at aquacultures integrated with agriculture systems in Egypt. The fish included in the study were apparently healthy, indicating they could act as reservoirs for potentially pathogenic bacteria in humans. Such resistant strains might have occurred in the aquaculture due to unregulated use of antibiotics to treat fish, or through agricultural sources, faecal contaminated tap water, or the workers. The resistant strains carry a variety of resistance Inc. plasmids which are similar between the strains from different sources. Most of these plasmids have a broad-host range and pose a great risk for the possible spread of resistance. Therefore, we recommend that studies on antimicrobial resistance should consider thorough, systematic studies of animals, fish, humans, tap water, and sewage, as all these factors are linked together. The current study has some limitations, including examination of integrated aquaculture from only one governorate in Egypt and the limited number of the tested carbapenemase genes (*bla*_KPC_, *bla*_NDM-1_, and *bla*_OXA-48_). Therefore, further work is required to elucidate the extent of antibiotic resistance in more integrated aquaculture from other governorates. Particularly, to screen for carbapenems and extended-spectrum-cephalosporins, with including a wide panel of carbapenemase genes.

## Supplementary information

**Additional file 1.**

## Data Availability

All data generated or analysed during this study are included in this published article.

## Abbreviations

βLPE: β-lactamase-producing *Enterobacteriaceae*
CAZ: Ceftazidime
CDC: Centre for Disease Control and Prevention
CEPH: Cephalosporins
CLSI: Clinical and Laboratory Standards Institute
CPE: Carbapenemase-producing *Enterobacteriaceae*
CRE: Carbapenem-resistant *Enterobacteriaceae*
CRO: Ceftriaxone
CTX: Cefotaxime
*ECC*: *Enterobacter cloacae complex*
EMB: Eosin methylene blue agar
ESBL: Extended-spectrum β-lactam
ETP: Ertapenem
FOX: Cefoxitin
Inc. plasmids: Incompatible plasmids
IPM: Imipenem
*K. pneumoniae*: *Klebsiella pneumoniae*
MEM: Meropenem
