# Supplementary material for: Emergence of β-lactamase- and carbapenemase- producing Enterobacteriaceae at integrated fish farms
Source: Antimicrob Resist Infect Control. 2020 May 19;9:67. doi: 10.1186/s13756-020-00736-3 (PMC7236517; doi:10.1186/s13756-020-00736-3)
Supplement: Supplementary file 1 — Additional file 1. [file 13756_2020_736_MOESM1_ESM.docx]

**S1: Sequences of the primers used in PCR.**

| **Genes** | **Primers** | **Sequences (**5`-3`) | **Size (bp) of PCR product** | **Ref.** |
| --- | --- | --- | --- | --- |
| ***bla*_Ctx-m-15_** | F  R | GCGATGGGCAGTACCAGTAA  TTACCCAGCGTCAGATTCCG | 392 | **[26]** |
| ***bla*_SHV_** | F  R | TCAGCGAAAAACACCTTG TCCCGCAGATAAATCACCA | 472 |  |
| ***bla*_TEM_** | F  R | ATGAGTATTCAACATTTCCG TTACCAATGCTTAATCAGTGAG | 861 |  |
| ***bla*_PER-1_** | F  R | ATGAATGTCATTATAAAAGC TTAATTTGGGCTTAGGG | 926 |  |
| ***bla*_OXA-1_** | F  R | TTTTCTGTTGTTTGGGTTTT  TTTCTTGGCTTTTATGCTTG | 427 | **[27]** |
| ***bla*_KPC_** | F  R | ATGTCACTGTATCGCCGTCT  TTTTCAGAGCCTTACTGCCC | 882 | **[29]** |
| ***bla*_OXA-48_** | F  R | TTGGTG GCATCGATTATCGG  GAGCACTTCTTTTGTGATGGC | 743 |  |
| ***bla*_NDM_** | F  R | GGTTTGGCGATCTGGTTTTC  CGGAATGGCTCATCACGATC | 621 |  |

**S2: Determination and typing of Incompatibility (Inc) plasmids carried by CRE isolates from fish (F), fishpond water inlets (P), tap water (T), outlet water (O), as well as hand swab (HS) and faecal samples (FS) from workers using PBRT-kits.**

| **Genotype*:* I) Carbapenemase genes**  ***a) bla*_KPC_** | |
| --- | --- |
| *E. coli*, F1 | IncF [FIA, FIB-KQ, FII (FIIS, FIIK)]; IncHI [HI2]; IncX [X3]; IncI complex [IncI1α, IncI2, IncB/O]. |
| *E. coli*, O1 | IncF [FIA]; IncHI [HI2]; IncX [X3]; IncA/C; IncN; IncN2; IncR |
| *K. pneumoniae*, O2 | IncF [FIA, FIB-KN, FIIK]; IncHI [HI1, HI2, HIB-M]; IncX [X1, X3]; IncI [I1α, I2]; IncA/C; IncP [P1α]; IncN, IncN2. |
| *ECC*, T3 | IncF [FIA, FII (FIIS, FIIK)]; IncHI [HI2]; IncX [X3]; IncI complex [IncI1α, IncK]; IncN2. |
| *ECC,* T4 | IncF [FII (FIIS, FIIK)]; IncHI [HI2]; IncX [X3]; IncI complex [IncI2, IncK]. |
| ***b) bla*_KPC_, *bla*_OXA-48_, *bla*_NDM_** | |
| *ECC*, F3 | **IncF** [FIB, FIIK)]; **IncHI** [HIB-M]; **IncX** [X2]; **IncI complex** [**IncI**1α, **IncI**1γ, **IncL**, **IncM**]; **Inc**N |
| *ECC*, FS3 | **IncF** [FIA, FIB, FII]; **IncHI** [HI2, HIB-M, FIB-M]; **IncX** [X1, X2]; **IncA/C**; **Inc**N |
| *ECC*, HS3 | **IncF** [FIB]; **IncHI** [HI1, HI2, HIB-M]; **IncX** [X1, X4]; **IncA/C**; **IncP** [P1α]; **Inc**N; **Inc**N2; **Inc**R |
| ***c) bla*_KPC_**, ***bla*_OXA-48_** | |
| *E. coli*, T1 | **IncF** [FIA, FIB, FIIS)]; **IncHI** [HI2]; **IncX** [X3]; **IncI complex** [**IncI**1α, **IncI**2, **IncL, IncM**, **IncK**] |
| ***d) bla*_KPC_**, ***bla*_NDM_** | |
| *K. pneumoniae*, T5 | **IncF** [FIA, FIB-KN, FII]; **IncHI** [HI1, HI2, HIB-M, FIB-M]; **IncX** [X1, X3, X4]; **IncI complex** [**IncI**1α, **IncI**2, **IncB/O**]; **IncA/C**; **IncP** [P1α]; **Inc**N, **Inc**N2; **Inc**R; **Inc**U; **Inc**T |
| *K. pneumoniae*, FS4 | **IncF** [FIA, FIB-KN, FIIK)]; **IncHI** [HI1, HI2, HIB-M]; **IncX** [X1, X3]; **IncI** [I1α, I2]; **IncA/C**; **IncP** [P1α]; **Inc**N |
|  | |
| **Genotype*:* II) Carbapenemase and β-lactamase genes**  ***a) bla*_KPC,_ *bla*_CTX-M_**, ***bla*_SHV,_ *bla*_TEM_**, ***bla*_PER-1_** | |
| *E. coli*, F2 | **IncF** [FIA, FIB-KQ]; **IncX** [X1, X3]; **IncI complex** [**IncI**1α, **IncM, IncB/O**]; **IncA/C**; **IncP** [P1α]; **IncW** |
| *E. coli*, P3 | **IncF** [FIA, FIB, FII (FIIS, FIIK)]; **IncHI** [HI2]; **IncI complex** [**IncI**1α, **IncI**2, **IncK**]; **IncP** [P1α]; **IncW** |
| *E. coli*, P5 | **IncHI** [FIB-M]; **IncX** [X3]; **IncI complex** [**IncI1**α, **IncB/O**]; **Inc**W; **Inc**R |
| *E. coli*, FS2 | **IncF** [FIA, FIB]; **IncHI** [HI1, HI2, HIB-M]; **IncX** [X1, X3]; **IncI complex** [**IncI**1γ, **IncM**]; **IncA/C**; **IncP** [P1α]; **IncW**; **IncR** |
| *K. pneumoniae*, FS5 | **IncF** [FIA, FIB-KN, FII (FIIS, FIIK)]; **IncHI** [HI1, HI2, HIB-M];  **IncX** [X1, X3]; **IncI complex** [**IncI**1α, **IncI**2, **IncM**, **IncB/O**]; **IncA/C**; **IncP** [P1α]; **Inc**N, **Inc**N2; **Inc**T; **IncW** |
| *K. pneumoniae*, FS6 | **IncF** [FIB-KN, FII]; **IncHI** [HI2, HIB-M]; **IncX** [X3]; **IncI complex** [**IncI**1α, **IncI**2, **IncM**]; **IncP** [P1α]; **IncU**; **IncT**; **IncW** |
| ***b) bla*_KPC,_ *bla*_CTX-M_**, ***bla*_SHV,_ *bla*_TEM_** | |
| *E. coli*, HS2 | **IncF** [FIB, FII (FIIS, FIIK)]; **IncHI** [HI2]; **IncI complex** [**IncI**1α, **IncB/O**]; **Inc**W |
| *E. coli*, HS4 | **IncF** [FII (FIIS, FIIK)]; **IncHI** [HI1, HI2]; **IncX** [X2, X3]; **IncI** [I1α]; **IncA/C**; **Inc**W; **Inc**U |
| *E. coli*, T2 | **IncF** [FIA, FIB, FIIK]; **IncHI** [FIB-M]; **IncX** [X1]; **IncI complex** [**IncI**1α, **IncB/O**]; **IncA/C; IncW** |
| ***c) bla*_KPC,_ *bla*_CTX-M_**, ***bla*_SHV_** | |
| *ECC*, P1 | **IncF** [FIA, FIB, FII]; **IncHI** [HI2]; **IncI complex** [**IncI**1α, **Inc**M]; **IncA/C**; **Inc**N |
| *K. pneumoniae*, F5 | **IncF** [FIA, FIB-KN, FIB-KQ), FII (FIIS, FIIK)]; **IncHI** [HIB-M]; **IncX** [X1, X3, X4]; **IncI** [I1α, I2]; IncW |
| *K. pneumoniae*, F6 | **IncF** [FIB-KN, FIIK)]; **IncHI** [HIB-M]; **IncX** [X2]; **IncI** [1α]; **Inc**U; **Inc**T |
| ***d) bla*_KPC,_ *bla*_NDM_**, ***bla*_CTX-M_**, ***bla*_SHV,_ *bla*_TEM_, *bla*_PER-1_** | |
| *ECC*, F4 | **IncF** [FIA, FII]; **IncHI** [HI1, HI2, HIB-M, FIB-M]; **IncX** [X1, X2, X3, X4]; **IncI complex** [**IncI**1α, **IncM**, **IncB/O**]; **IncA/C**; **IncP** [P1α]; **Inc**W |
| ***e) bla*_KPC,_ *bla*_NDM_**, ***bla*_CTX-M_**, ***bla*_SHV,_ *bla*_TEM_** | |
| *K. pneumoniae*, T6 | **IncF** [FIA, FIB (FIB-KQ), FII (FIIK)]; **IncHI** [HI1, HIB-M]; **IncX** [X2, X3]; **IncI complex** [**IncI**1α, **IncM**]; **IncA/C**; **IncP** [P1α]; **IncW** |
| ***f) bla*_KPC,_ *bla*_OXA-48_**, ***bla*_CTX-M_**, ***bla*_SHV,_ *bla*_TEM_ *bla*_OXA-1_, *bla*_PER-1_** | |
| *E. coli*, O3 | **IncF** [FIA, FIB (FIB-KQ), FII (FIIS)]; **IncHI** [HI1, HI2]; **IncX** [X2]; **IncI complex** [**IncI**1α, **IncI**1γ, **IncB/O**]; **IncA/C**; **Inc**N; **Inc**N2; **Inc**W; **Inc**R |
| ***g) bla*_KPC,_ *bla*_OXA-48_**, ***bla*_CTX-M_**, ***bla*_SHV_** | |
| *ECC*, O4 | **IncF** [FIA, FIB, FII (FIIK)]; **IncHI** [HI1, HI2, HIB-M]; **IncX** [X1, X3]; **IncM**; **IncA/C**; **IncP** [P1α] |
| ***h) bla*_KPC,_ *bla*_OXA-48_**, ***bla*_NDM_**, ***bla*_CTX-M_**, ***bla*_SHV,_ *bla*_TEM_** | |
| *E. coli*, HS1 | **IncF** [FIA, FII (FIIS, FIIK)]; **IncHI** [HIB-M]; **IncX** [X3]; **IncI complex** [**IncI**1α, **IncK**, **IncL**, **IncM**]; **IncN; IncW** |
| *E. coli*, HS5 | **IncF** [FIA, FIB, FII (FIIS, FIIK)]; **IncHI** [HI2]; **IncX** [X1, X3]; **IncI complex** [**IncI**1α, **IncI**1γ, **IncM**, **IncB/O**]; **IncA/C**; **IncP** [P1α] |
| ***i) bla*_KPC,_ *bla*_OXA-48_**, ***bla*_NDM_**, ***bla*_CTX-M_**, ***bla*_SHV,_ *bla*_TEM,_ *bla*_PER-1_** | |
| *E. coli*, FS1 | **IncF** [FIA, FIB, FII (FIIS, FIIK)]; **IncHI** [HI2, HIB-M]]; **IncX** [X1, X3]; **IncI complex** [**IncI**1α, **IncI**2, **IncB/O**; **IncL; IncM**]; **IncP** [P1α]; **Inc**W |
